# 3,5-Bis(3-methyl­imidazolium-1-ylmeth­yl)toluene bis­(hexa­fluoro­phosphate)

**DOI:** 10.1107/S1600536810003739

**Published:** 2010-02-06

**Authors:** Abbas Washeel, Rosenani A. Haque, Siang Guan Teoh, Chin Sing Yeap, Hoong-Kun Fun

**Affiliations:** a School of Chemical Science, Universiti Sains Malaysia, 11800 USM, Penang, Malaysia; b X-ray Crystallography Unit, School of Physics, Universiti Sains Malaysia, 11800 USM, Penang, Malaysia

## Abstract

The asymmetric unit of the title *N*-heterocyclic carbene compound, C_17_H_22_N_4_^2+^·2PF_6_^−^, consists of one *N*-heterocyclic carbene dication and two hexa­fluoro­phosphate anions. The two imidazole rings are twisted away from but to the same side of the central toluene ring, making dihedral angles of 76.69 (7) and 78.03 (7)° with the central ring. In the crystal, the components are linked by C—H⋯F interactions, generating a three-dimensional network.

## Related literature

For background to *N*-heterocyclic carbenes, see: Wanzlick & Schönherr (1968 ▶); Öfele (1968 ▶); Arduengo *et al.* (1991 ▶). For applications of *N*-heterocyclic carbene derivatives, see: Meyer *et al.* (2009 ▶); Ray *et al.* (2007 ▶); Medvetz *et al.* (2008 ▶). For a related structure, see: Jiang (2009 ▶). For the synthesis, see; Dias & Jin (1994 ▶). For the stability of the temperature controller used for the data collection, see: Cosier & Glazer (1986 ▶).
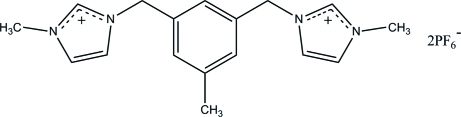


## Experimental

### 

#### Crystal data

C_17_H_22_N_4_^2+^·2PF_6_^−^
*M* *_r_* = 572.33
Monoclinic, 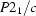

*a* = 6.1289 (1) Å
*b* = 19.0139 (4) Å
*c* = 20.1770 (4) Å
β = 97.390 (1)°
*V* = 2331.78 (8) Å^3^
*Z* = 4
Mo *K*α radiation
μ = 0.30 mm^−1^
*T* = 100 K
0.53 × 0.28 × 0.20 mm

#### Data collection

Bruker SMART APEXII CCD area-detector diffractometer
Absorption correction: multi-scan (*SADABS*; Bruker, 2009 ▶) *T*_min_ = 0.858, *T*_max_ = 0.942
42881 measured reflections
11275 independent reflections
7988 reflections with *I* > 2σ(*I*)
*R*_int_ = 0.034

#### Refinement

*R*[*F* ^2^ > 2σ(*F* ^2^)] = 0.052
*wR*(*F* ^2^) = 0.149
*S* = 1.03
11275 reflections
319 parameters
H-atom parameters constrained
Δρ_max_ = 0.62 e Å^−3^
Δρ_min_ = −0.44 e Å^−3^

### 

Data collection: *APEX2* (Bruker, 2009 ▶); cell refinement: *SAINT* (Bruker, 2009 ▶); data reduction: *SAINT*; program(s) used to solve structure: *SHELXTL* (Sheldrick, 2008 ▶); program(s) used to refine structure: *SHELXTL*; molecular graphics: *SHELXTL*; software used to prepare material for publication: *SHELXTL* and *PLATON* (Spek, 2009 ▶).

## Supplementary Material

Crystal structure: contains datablocks global, I. DOI: 10.1107/S1600536810003739/tk2620sup1.cif

Structure factors: contains datablocks I. DOI: 10.1107/S1600536810003739/tk2620Isup2.hkl

Additional supplementary materials:  crystallographic information; 3D view; checkCIF report

## Figures and Tables

**Table 1:** Hydrogen-bond geometry (Å, °)

*D*—H⋯*A*	*D*—H	H⋯*A*	*D*⋯*A*	*D*—H⋯*A*
C1—H1*A*⋯F2^i^	0.93	2.47	3.3431 (16)	157
C5—H5*A*⋯F8^ii^	0.93	2.39	3.2686 (16)	157
C8—H8*A*⋯F12^ii^	0.93	2.40	3.2808 (16)	158
C9—H9*A*⋯F4^i^	0.93	2.41	3.2513 (19)	151
C10—H10*A*⋯F4^iii^	0.93	2.38	3.2967 (17)	169
C12—H12*B*⋯F5^iv^	0.97	2.42	3.3040 (18)	150
C14—H14*A*⋯F8^iii^	0.93	2.55	3.283 (2)	136
C14—H14*A*⋯F10^iii^	0.93	2.53	3.3050 (19)	141
C15—H15*A*⋯F7^v^	0.93	2.50	3.2568 (19)	139
C16—H16*B*⋯F9^vi^	0.96	2.48	3.135 (2)	125
C16—H16*C*⋯F2	0.96	2.48	3.314 (2)	146
C17—H17*C*⋯F11	0.96	2.42	3.3563 (18)	166

Symmetry codes: (i) 
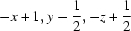
; (ii) 
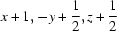
; (iii) 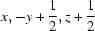; (iv) 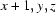; (v) 
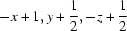
; (vi) 
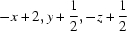
.

**Fig. 1.**
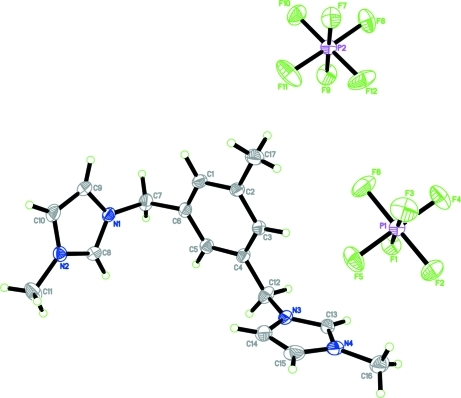
The molecular structure of the title compound with 50% probability ellipsoids for non-H atoms.

**Fig. 2.**
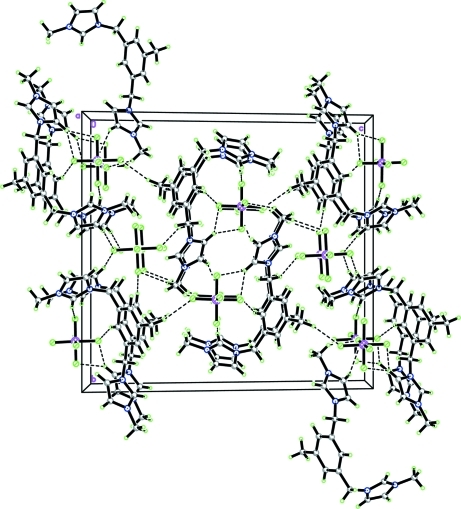
The crystal packing of the title compound, viewed down the a axis, showing the molecules are linked a 3-D network. Intermolecular hydrogen bonds are shown as dashed lines.

## supplementary crystallographic information

### Comment

*N*-Heterocyclic carbenes (NHCs) were first pioneered by Wanzlick and Öfele in 1968 (Wanzlick & Schönherr, 1968; Öfele, 1968). After the isolation of the first stable crystalline carbene by Arduengo in 1991, the research area of NHCs has continued to grow (Arduengo *et al.*, 1991). NHCs are an important class of ligands and their metal complexes are important in homogeneous catalysis for a wide range of reactions including C–C coupling reactions, olefin metathesis, hydroformylation, and polymerization reactions (Meyer *et al.*, 2009). The biological activities of many of these complexes were confirmed (Ray *et al.*, 2007) and the silver complexes derived from 4,5-dichloro-1*H*-imidazole were found to be active against human cancer cell lines (Medvetz *et al.*, 2008).

The asymmetric unit of the title compound consists of one *N*-heterocyclic carbene dication and two hexafluorophosphate anions (Fig. 1). The geometric parameters are comparable to those of a related structure (Jiang, 2009). Each of the imidazole rings (N1–C8–N2–C10–C9 and N3–C13–N4–C15–C14) is planar with a maximum deviation of 0.003 (1) Å (for N1) and 0.002 (2) Å (for C14 & C15), respectively. The two imidazole rings are twisted away from the central toluene ring but are orientated to the same side of the ring, making dihedral angles of 76.69 (7) and 78.03 (7)° with the central ring, respectively. The two hexafluorophosphate anions link the ions into a three-dimensional network *via* intermolecular C—H···F hydrogen bonds (Fig. 2, Table 1).

### Experimental

The title compound was prepared following the procedure of Dias & Jin (1994). To a stirred solution of 3,5-bis(bromomethyl)toluene (1.0 g, 3.6 mmol) in 1,4-dioxane (20 ml), 1-methylimidazole (0.9 g, 7.2 mmol) was added. The mixture was refluxed at 373 K for 24 h. The sticky product was isolated by decantation, then washed with diethyl ether (2x3 ml). KPF_6_ (1.3 g, 7.2 mmol) in methanol (20 ml) was added with stirring for 1 h. Then the mixture left standing overnight. The beige precipitate was collected and washed with distilled water and recrystallised from hot methanol. The yield was 1.5 g (72.88 %), m.pt.: 467-469 K. Crystals were obtained by slow evaporation of the salt solution in acetonitrile at low temperature.

### Refinement

All H atoms were positioned geometrically and refined using a riding model, with C–H = 0.93-0.97 Å and *U*_iso_(H) = 1.2 or 1.5 *U*_eq_(C). The rotating group model was applied for the methyl groups.

### Crystal data

**Table:**

C_17_H_22_N_4_^2+^·2PF_6_^−^	*F*(000) = 1160
*M**_r_* = 572.33	*D*_x_ = 1.630 Mg m^−^^3^
Monoclinic, *P*2_1_/*c*	Mo *K*α radiation, λ = 0.71073 Å
Hall symbol: -P 2ybc	Cell parameters from 9980 reflections
*a* = 6.1289 (1) Å	θ = 2.3–33.1°
*b* = 19.0139 (4) Å	µ = 0.30 mm^−^^1^
*c* = 20.1770 (4) Å	*T* = 100 K
β = 97.390 (1)°	Block, colourless
*V* = 2331.78 (8) Å^3^	0.53 × 0.28 × 0.20 mm
*Z* = 4	

### Data collection

**Table:**

Bruker SMART APEXII CCD area-detector diffractometer	11275 independent reflections
Radiation source: fine-focus sealed tube	7988 reflections with *I* > 2σ(*I*)
graphite	*R*_int_ = 0.034
φ and ω scans	θ_max_ = 36.4°, θ_min_ = 2.0°
Absorption correction: multi-scan (*SADABS*; Bruker, 2009)	*h* = −9→10
*T*_min_ = 0.858, *T*_max_ = 0.942	*k* = −31→31
42881 measured reflections	*l* = −33→33

### Refinement

**Table:**

Refinement on *F*^2^	Primary atom site location: structure-invariant direct methods
Least-squares matrix: full	Secondary atom site location: difference Fourier map
*R*[*F*^2^ > 2σ(*F*^2^)] = 0.052	Hydrogen site location: inferred from neighbouring sites
*wR*(*F*^2^) = 0.149	H-atom parameters constrained
*S* = 1.03	*w* = 1/[σ^2^(*F*_o_^2^) + (0.0703*P*)^2^ + 0.7107*P*] where *P* = (*F*_o_^2^ + 2*F*_c_^2^)/3
11275 reflections	(Δ/σ)_max_ = 0.001
319 parameters	Δρ_max_ = 0.62 e Å^−^^3^
0 restraints	Δρ_min_ = −0.44 e Å^−^^3^

### Special details

**Table:**

Experimental. The crystal was placed in the cold stream of an Oxford Cryosystems Cobra open-flow nitrogen cryostat (Cosier & Glazer, 1986) operating at 100.0 (1) K.
Geometry. All esds (except the esd in the dihedral angle between two l.s. planes) are estimated using the full covariance matrix. The cell esds are taken into account individually in the estimation of esds in distances, angles and torsion angles; correlations between esds in cell parameters are only used when they are defined by crystal symmetry. An approximate (isotropic) treatment of cell esds is used for estimating esds involving l.s. planes.
Refinement. Refinement of *F*^2^ against ALL reflections. The weighted *R*-factor wR and goodness of fit *S* are based on *F*^2^, conventional *R*-factors *R* are based on *F*, with *F* set to zero for negative *F*^2^. The threshold expression of *F*^2^ > σ(*F*^2^) is used only for calculating *R*-factors(gt) etc. and is not relevant to the choice of reflections for refinement. *R*-factors based on *F*^2^ are statistically about twice as large as those based on *F*, and *R*- factors based on ALL data will be even larger.

### Fractional atomic coordinates and isotropic or equivalent isotropic displacement parameters (Å^2^)

**Table:**

	*x*	*y*	*z*	*U*_iso_*/*U*_eq_	
N1	1.09705 (19)	0.13322 (5)	0.47609 (5)	0.02147 (19)	
N2	1.01744 (18)	0.14740 (6)	0.57634 (5)	0.02158 (19)	
N3	1.07523 (19)	0.45850 (6)	0.36551 (5)	0.0224 (2)	
N4	0.89594 (19)	0.55547 (6)	0.34480 (6)	0.0241 (2)	
C1	0.8951 (2)	0.20094 (7)	0.33537 (6)	0.0244 (2)	
H1A	0.8258	0.1574	0.3299	0.029*	
C2	0.7957 (2)	0.25985 (7)	0.30300 (6)	0.0229 (2)	
C3	0.9031 (2)	0.32451 (7)	0.31150 (6)	0.0220 (2)	
H3A	0.8388	0.3642	0.2904	0.026*	
C4	1.1060 (2)	0.33027 (7)	0.35138 (6)	0.0224 (2)	
C5	1.2019 (2)	0.27104 (7)	0.38343 (6)	0.0230 (2)	
H5A	1.3370	0.2747	0.4101	0.028*	
C6	1.0960 (2)	0.20638 (7)	0.37567 (6)	0.0229 (2)	
C7	1.1966 (3)	0.14348 (7)	0.41401 (7)	0.0284 (3)	
H7A	1.1736	0.1017	0.3864	0.034*	
H7B	1.3539	0.1506	0.4249	0.034*	
C8	1.1522 (2)	0.16744 (6)	0.53334 (6)	0.0216 (2)	
H8A	1.2659	0.1999	0.5418	0.026*	
C9	0.9202 (3)	0.09042 (7)	0.48282 (7)	0.0287 (3)	
H9A	0.8481	0.0610	0.4503	0.034*	
C10	0.8715 (2)	0.09935 (8)	0.54570 (7)	0.0293 (3)	
H10A	0.7596	0.0770	0.5647	0.035*	
C11	1.0210 (3)	0.17284 (9)	0.64499 (7)	0.0326 (3)	
H11A	1.1495	0.2013	0.6568	0.049*	
H11B	1.0243	0.1335	0.6749	0.049*	
H11C	0.8917	0.2004	0.6483	0.049*	
C12	1.2268 (2)	0.39980 (7)	0.36005 (8)	0.0286 (3)	
H12A	1.3332	0.3979	0.4000	0.034*	
H12B	1.3064	0.4076	0.3221	0.034*	
C13	1.0490 (2)	0.51400 (7)	0.32521 (6)	0.0215 (2)	
H13A	1.1257	0.5224	0.2891	0.026*	
C14	0.9316 (3)	0.46493 (8)	0.41259 (7)	0.0310 (3)	
H14A	0.9148	0.4333	0.4467	0.037*	
C15	0.8204 (3)	0.52554 (8)	0.39983 (8)	0.0316 (3)	
H15A	0.7131	0.5438	0.4236	0.038*	
C16	0.8185 (3)	0.62160 (8)	0.31312 (8)	0.0360 (3)	
H16A	0.9219	0.6376	0.2845	0.054*	
H16B	0.8050	0.6563	0.3469	0.054*	
H16C	0.6778	0.6143	0.2871	0.054*	
C17	0.5767 (2)	0.25388 (8)	0.26001 (7)	0.0307 (3)	
H17A	0.5086	0.2994	0.2553	0.046*	
H17B	0.4836	0.2222	0.2806	0.046*	
H17C	0.5982	0.2362	0.2168	0.046*	
P1	0.48424 (5)	0.488905 (18)	0.175041 (16)	0.02100 (7)	
F1	0.74701 (13)	0.49029 (5)	0.18064 (5)	0.03038 (18)	
F2	0.48256 (17)	0.57311 (5)	0.17898 (5)	0.0381 (2)	
F3	0.22094 (15)	0.48912 (6)	0.16927 (6)	0.0434 (2)	
F4	0.46926 (17)	0.49406 (6)	0.09530 (4)	0.0407 (2)	
F5	0.50201 (18)	0.48512 (7)	0.25471 (5)	0.0442 (3)	
F6	0.48927 (19)	0.40533 (5)	0.16983 (6)	0.0488 (3)	
P2	0.66006 (5)	0.168232 (18)	0.043019 (17)	0.02251 (8)	
F7	0.39775 (14)	0.15921 (5)	0.03209 (5)	0.0366 (2)	
F8	0.66196 (17)	0.16954 (6)	−0.03614 (5)	0.0412 (2)	
F9	0.92024 (15)	0.17795 (6)	0.05369 (6)	0.0421 (2)	
F10	0.69001 (18)	0.08453 (5)	0.04230 (6)	0.0420 (2)	
F11	0.6604 (2)	0.16457 (8)	0.12203 (5)	0.0575 (4)	
F12	0.62782 (18)	0.25150 (5)	0.04312 (7)	0.0552 (3)	

### Atomic displacement parameters (Å^2^)

**Table:**

	*U*^11^	*U*^22^	*U*^33^	*U*^12^	*U*^13^	*U*^23^
N1	0.0274 (5)	0.0155 (4)	0.0224 (4)	0.0010 (4)	0.0065 (4)	0.0017 (4)
N2	0.0240 (5)	0.0208 (5)	0.0200 (4)	0.0028 (4)	0.0029 (4)	0.0019 (4)
N3	0.0262 (5)	0.0190 (5)	0.0223 (4)	−0.0057 (4)	0.0044 (4)	0.0003 (4)
N4	0.0248 (5)	0.0214 (5)	0.0254 (5)	−0.0017 (4)	0.0010 (4)	−0.0041 (4)
C1	0.0336 (7)	0.0184 (5)	0.0230 (5)	−0.0047 (5)	0.0101 (5)	−0.0033 (4)
C2	0.0285 (6)	0.0220 (5)	0.0192 (5)	−0.0055 (4)	0.0072 (4)	−0.0028 (4)
C3	0.0268 (6)	0.0202 (5)	0.0195 (5)	−0.0020 (4)	0.0050 (4)	0.0006 (4)
C4	0.0265 (6)	0.0191 (5)	0.0223 (5)	−0.0025 (4)	0.0060 (4)	0.0007 (4)
C5	0.0251 (6)	0.0225 (5)	0.0220 (5)	0.0006 (4)	0.0060 (4)	0.0007 (4)
C6	0.0317 (6)	0.0186 (5)	0.0205 (5)	0.0023 (4)	0.0115 (4)	0.0002 (4)
C7	0.0401 (7)	0.0207 (6)	0.0275 (6)	0.0084 (5)	0.0158 (5)	0.0025 (5)
C8	0.0209 (5)	0.0187 (5)	0.0253 (5)	−0.0008 (4)	0.0034 (4)	−0.0007 (4)
C9	0.0364 (7)	0.0223 (6)	0.0268 (6)	−0.0105 (5)	0.0013 (5)	0.0016 (5)
C10	0.0290 (6)	0.0300 (7)	0.0292 (6)	−0.0090 (5)	0.0053 (5)	0.0062 (5)
C11	0.0418 (8)	0.0350 (7)	0.0211 (5)	0.0130 (6)	0.0048 (5)	−0.0007 (5)
C12	0.0238 (6)	0.0216 (6)	0.0402 (7)	−0.0035 (4)	0.0031 (5)	0.0041 (5)
C13	0.0232 (5)	0.0215 (5)	0.0201 (5)	−0.0031 (4)	0.0037 (4)	0.0003 (4)
C14	0.0437 (8)	0.0272 (6)	0.0247 (6)	−0.0118 (6)	0.0145 (5)	−0.0026 (5)
C15	0.0350 (7)	0.0304 (7)	0.0320 (6)	−0.0078 (5)	0.0146 (6)	−0.0107 (5)
C16	0.0386 (8)	0.0261 (7)	0.0395 (8)	0.0054 (6)	−0.0089 (6)	−0.0022 (6)
C17	0.0309 (7)	0.0333 (7)	0.0275 (6)	−0.0074 (5)	0.0022 (5)	−0.0039 (5)
P1	0.01773 (14)	0.02285 (15)	0.02274 (14)	0.00107 (10)	0.00376 (11)	0.00061 (11)
F1	0.0179 (3)	0.0379 (5)	0.0356 (4)	0.0027 (3)	0.0042 (3)	0.0001 (4)
F2	0.0392 (5)	0.0242 (4)	0.0517 (6)	0.0057 (4)	0.0091 (4)	−0.0031 (4)
F3	0.0176 (4)	0.0592 (7)	0.0538 (6)	−0.0036 (4)	0.0061 (4)	−0.0061 (5)
F4	0.0399 (5)	0.0593 (6)	0.0223 (4)	0.0208 (5)	0.0018 (4)	−0.0017 (4)
F5	0.0407 (5)	0.0687 (8)	0.0247 (4)	−0.0091 (5)	0.0100 (4)	0.0080 (4)
F6	0.0449 (6)	0.0229 (4)	0.0752 (8)	−0.0043 (4)	−0.0051 (5)	0.0019 (5)
P2	0.01778 (14)	0.02462 (16)	0.02537 (15)	0.00125 (11)	0.00367 (11)	−0.00180 (12)
F7	0.0190 (4)	0.0468 (5)	0.0443 (5)	−0.0040 (4)	0.0055 (3)	0.0089 (4)
F8	0.0350 (5)	0.0598 (7)	0.0299 (4)	0.0093 (4)	0.0084 (4)	0.0100 (4)
F9	0.0178 (4)	0.0478 (6)	0.0593 (6)	0.0006 (4)	−0.0010 (4)	0.0095 (5)
F10	0.0447 (6)	0.0269 (4)	0.0538 (6)	0.0047 (4)	0.0039 (5)	0.0042 (4)
F11	0.0468 (6)	0.1009 (11)	0.0251 (4)	0.0206 (6)	0.0064 (4)	−0.0085 (5)
F12	0.0356 (5)	0.0249 (5)	0.1015 (10)	0.0045 (4)	−0.0052 (6)	−0.0120 (5)

### Geometric parameters (Å, °)

**Table:**

N1—C8	1.3310 (16)	C10—H10A	0.9300
N1—C9	1.3759 (18)	C11—H11A	0.9600
N1—C7	1.4752 (17)	C11—H11B	0.9600
N2—C8	1.3284 (17)	C11—H11C	0.9600
N2—C10	1.3691 (18)	C12—H12A	0.9700
N2—C11	1.4647 (17)	C12—H12B	0.9700
N3—C13	1.3294 (16)	C13—H13A	0.9300
N3—C14	1.3810 (18)	C14—C15	1.347 (2)
N3—C12	1.4652 (18)	C14—H14A	0.9300
N4—C13	1.3242 (17)	C15—H15A	0.9300
N4—C15	1.3795 (19)	C16—H16A	0.9600
N4—C16	1.4618 (19)	C16—H16B	0.9600
C1—C6	1.389 (2)	C16—H16C	0.9600
C1—C2	1.3965 (19)	C17—H17A	0.9600
C1—H1A	0.9300	C17—H17B	0.9600
C2—C3	1.3945 (17)	C17—H17C	0.9600
C2—C17	1.5059 (19)	P1—F6	1.5932 (10)
C3—C4	1.3959 (18)	P1—F5	1.5988 (10)
C3—H3A	0.9300	P1—F1	1.6001 (9)
C4—C5	1.3907 (18)	P1—F4	1.6027 (9)
C4—C12	1.5143 (18)	P1—F3	1.6029 (10)
C5—C6	1.3897 (18)	P1—F2	1.6031 (10)
C5—H5A	0.9300	P2—F9	1.5921 (10)
C6—C7	1.5123 (18)	P2—F11	1.5955 (11)
C7—H7A	0.9700	P2—F12	1.5957 (11)
C7—H7B	0.9700	P2—F8	1.5989 (10)
C8—H8A	0.9300	P2—F10	1.6024 (10)
C9—C10	1.351 (2)	P2—F7	1.6036 (9)
C9—H9A	0.9300		
			
C8—N1—C9	108.59 (11)	N3—C12—H12B	109.3
C8—N1—C7	125.85 (12)	C4—C12—H12B	109.3
C9—N1—C7	125.40 (12)	H12A—C12—H12B	108.0
C8—N2—C10	108.76 (11)	N4—C13—N3	108.95 (11)
C8—N2—C11	126.01 (12)	N4—C13—H13A	125.5
C10—N2—C11	125.23 (12)	N3—C13—H13A	125.5
C13—N3—C14	108.23 (12)	C15—C14—N3	107.21 (12)
C13—N3—C12	125.92 (12)	C15—C14—H14A	126.4
C14—N3—C12	125.84 (12)	N3—C14—H14A	126.4
C13—N4—C15	108.57 (12)	C14—C15—N4	107.04 (13)
C13—N4—C16	125.75 (13)	C14—C15—H15A	126.5
C15—N4—C16	125.68 (13)	N4—C15—H15A	126.5
C6—C1—C2	120.95 (12)	N4—C16—H16A	109.5
C6—C1—H1A	119.5	N4—C16—H16B	109.5
C2—C1—H1A	119.5	H16A—C16—H16B	109.5
C3—C2—C1	118.63 (12)	N4—C16—H16C	109.5
C3—C2—C17	120.59 (12)	H16A—C16—H16C	109.5
C1—C2—C17	120.78 (12)	H16B—C16—H16C	109.5
C2—C3—C4	120.75 (12)	C2—C17—H17A	109.5
C2—C3—H3A	119.6	C2—C17—H17B	109.5
C4—C3—H3A	119.6	H17A—C17—H17B	109.5
C5—C4—C3	119.76 (12)	C2—C17—H17C	109.5
C5—C4—C12	118.82 (12)	H17A—C17—H17C	109.5
C3—C4—C12	121.41 (12)	H17B—C17—H17C	109.5
C6—C5—C4	120.04 (12)	F6—P1—F5	91.26 (7)
C6—C5—H5A	120.0	F6—P1—F1	89.61 (6)
C4—C5—H5A	120.0	F5—P1—F1	89.54 (5)
C1—C6—C5	119.86 (12)	F6—P1—F4	89.65 (6)
C1—C6—C7	120.87 (12)	F5—P1—F4	178.89 (7)
C5—C6—C7	119.21 (13)	F1—P1—F4	89.83 (5)
N1—C7—C6	110.66 (10)	F6—P1—F3	91.47 (6)
N1—C7—H7A	109.5	F5—P1—F3	90.62 (6)
C6—C7—H7A	109.5	F1—P1—F3	178.90 (6)
N1—C7—H7B	109.5	F4—P1—F3	89.99 (6)
C6—C7—H7B	109.5	F6—P1—F2	178.73 (7)
H7A—C7—H7B	108.1	F5—P1—F2	89.72 (6)
N2—C8—N1	108.46 (11)	F1—P1—F2	89.59 (5)
N2—C8—H8A	125.8	F4—P1—F2	89.36 (6)
N1—C8—H8A	125.8	F3—P1—F2	89.32 (6)
C10—C9—N1	106.89 (12)	F9—P2—F11	89.85 (6)
C10—C9—H9A	126.6	F9—P2—F12	90.39 (6)
N1—C9—H9A	126.6	F11—P2—F12	91.50 (8)
C9—C10—N2	107.30 (12)	F9—P2—F8	89.86 (6)
C9—C10—H10A	126.4	F11—P2—F8	178.32 (7)
N2—C10—H10A	126.4	F12—P2—F8	90.15 (7)
N2—C11—H11A	109.5	F9—P2—F10	90.15 (6)
N2—C11—H11B	109.5	F11—P2—F10	88.87 (7)
H11A—C11—H11B	109.5	F12—P2—F10	179.35 (7)
N2—C11—H11C	109.5	F8—P2—F10	89.47 (6)
H11A—C11—H11C	109.5	F9—P2—F7	179.46 (6)
H11B—C11—H11C	109.5	F11—P2—F7	90.30 (6)
N3—C12—C4	111.62 (11)	F12—P2—F7	89.09 (6)
N3—C12—H12A	109.3	F8—P2—F7	90.02 (6)
C4—C12—H12A	109.3	F10—P2—F7	90.37 (6)
			
C6—C1—C2—C3	0.45 (19)	C7—N1—C8—N2	175.98 (11)
C6—C1—C2—C17	−179.27 (12)	C8—N1—C9—C10	−0.46 (16)
C1—C2—C3—C4	0.19 (19)	C7—N1—C9—C10	−175.99 (12)
C17—C2—C3—C4	179.91 (12)	N1—C9—C10—N2	0.27 (17)
C2—C3—C4—C5	−0.45 (19)	C8—N2—C10—C9	0.01 (16)
C2—C3—C4—C12	178.79 (12)	C11—N2—C10—C9	179.28 (13)
C3—C4—C5—C6	0.08 (19)	C13—N3—C12—C4	−120.97 (14)
C12—C4—C5—C6	−179.19 (12)	C14—N3—C12—C4	57.56 (18)
C2—C1—C6—C5	−0.83 (19)	C5—C4—C12—N3	−141.60 (12)
C2—C1—C6—C7	176.25 (11)	C3—C4—C12—N3	39.15 (18)
C4—C5—C6—C1	0.56 (19)	C15—N4—C13—N3	−0.04 (15)
C4—C5—C6—C7	−176.57 (11)	C16—N4—C13—N3	−179.56 (12)
C8—N1—C7—C6	−81.82 (17)	C14—N3—C13—N4	0.27 (14)
C9—N1—C7—C6	92.95 (16)	C12—N3—C13—N4	179.02 (11)
C1—C6—C7—N1	−80.96 (15)	C13—N3—C14—C15	−0.40 (16)
C5—C6—C7—N1	96.14 (15)	C12—N3—C14—C15	−179.15 (12)
C10—N2—C8—N1	−0.30 (15)	N3—C14—C15—N4	0.37 (16)
C11—N2—C8—N1	−179.56 (12)	C13—N4—C15—C14	−0.21 (16)
C9—N1—C8—N2	0.47 (15)	C16—N4—C15—C14	179.31 (13)

### Hydrogen-bond geometry (Å, °)

**Table:**

*D*—H···*A*	*D*—H	H···*A*	*D*···*A*	*D*—H···*A*
C1—H1A···F2^i^	0.93	2.47	3.3431 (16)	157
C5—H5A···F8^ii^	0.93	2.39	3.2686 (16)	157
C8—H8A···F12^ii^	0.93	2.40	3.2808 (16)	158
C9—H9A···F4^i^	0.93	2.41	3.2513 (19)	151
C10—H10A···F4^iii^	0.93	2.38	3.2967 (17)	169
C12—H12B···F5^iv^	0.97	2.42	3.3040 (18)	150
C14—H14A···F8^iii^	0.93	2.55	3.283 (2)	136
C14—H14A···F10^iii^	0.93	2.53	3.3050 (19)	141
C15—H15A···F7^v^	0.93	2.50	3.2568 (19)	139
C16—H16B···F9^vi^	0.96	2.48	3.135 (2)	125
C16—H16C···F2	0.96	2.48	3.314 (2)	146
C17—H17C···F11	0.96	2.42	3.3563 (18)	166

Symmetry codes: (i) −*x*+1, *y*−1/2, −*z*+1/2; (ii) *x*+1, −*y*+1/2, *z*+1/2; (iii) *x*, −*y*+1/2, *z*+1/2; (iv) *x*+1, *y*, *z*; (v) −*x*+1, *y*+1/2, −*z*+1/2; (vi) −*x*+2, *y*+1/2, −*z*+1/2.
